# Tiny tools closing the gap: nanobodies in research and therapy

**DOI:** 10.1152/function.107.2025

**Published:** 2026-02-02

**Authors:** Mustafa Abdellatif, Jyotirmoy Rajurkar, Heinrich Leonhardt

**Affiliations:** Faculty of Biology, Human Biology and BioImaging, LMU Munich, Martinsried, Germany

**Keywords:** antibodies, cell biology, microscopy, nanobodies, targeted therapy

## Abstract

Nanobodies, also known as single-domain antibodies, have become powerful tools in research, diagnosis, and therapy. Sourced from the heavy chain of camelid heavy chain-only antibodies, this domain retains many important characteristics of full-length antibodies while being ∼10 times smaller in molecular weight. Nanobody discovery has seen expansive development over the recent past, through both conventional antigen-exposed and completely synthetic repertoires. Along these lines, binding properties of candidates can be evolved by subsequent mutation and selection cycles to adjust their specificity and avidity. Due to their small size and compact structure, nanobodies can reach cryptic sites not accessible to conventional antibodies and also show superior tissue penetration. This penetrance, alongside their ease of handling, has made nanobodies ideal candidates for a myriad of immunotherapeutic and drug delivery applications. Furthermore, their small size imparts minimal linkage errors when conjugated to a fluorophore, making nanobodies ideal tools for high-resolution imaging techniques. Most importantly, nanobodies can be stably expressed in living cells to bind, block, or modify intracellular targets, enabling study of proteins in a native context at unprecedented detail. In this review, we present the latest developments in nanobody technology and discuss applications in bioimaging, therapy, and intracellular protein study.

## Graphical abstract

**Figure:**
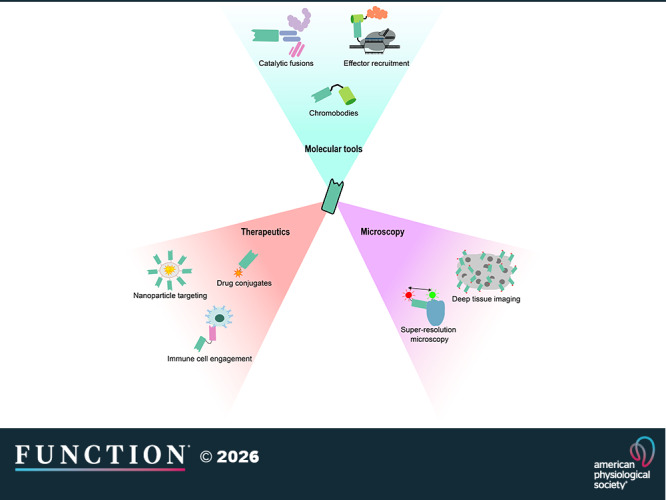

## INTRODUCTION

Over the past two decades, the realm of targeted therapy has seen momentous progress via developments in the expressing, equipping, and engineering of disease-specific antibodies. This was namely brought about by advances in discovery/production systems, conjugation chemistries, and genetic manipulation, taking antibody-based therapeutics to the forefront of precision medicine. No longer restricted to full-length antibodies, many modern therapeutics consist of shortened antibody formats such as single-chain variable (scFv) or antigen binding (Fab) fragments, which consist of artificially shortened derivatives that maintain the variable light-variable heavy domain (VL-VH) paratope interface of the parent antibody.

In 1993, however, heavy chain-only antibodies (hcAbs) were discovered in camelids (1). As the name suggests, these binders possess fully functional paratopes in the absence of the constant and variable light domains. The variable heavy domain (VHH) of a hcAb could thus be isolated, cloned, and recombinantly produced to yield the smallest naturally derived binding domain ever known. This was the birth of the nanobody: a structurally simple, easy-to-produce, and profoundly stable antibody fragment that opened the doors to applications where full-length antibodies and their derivatives fall short. The potential of the nanobody was widely and immediately recognized, with the past three decades witnessing an emphatic expansion of the nanobody-based toolbox.

The lack of VL pairing, facilitated by hallmark substitutions in the framework 2 (FR2) interface, rendered nanobodies a captivating tool for the generation of large in vitro display libraries, whereas their single-domain nature enabled scalable and cheap expression in *Escherichia coli* systems. This contrasts full-length antibodies that suffer from complex assembly, hindering both display efficiency and expressibility. The tolerance of nanobodies to reducing environments and their consequent intracellular stability has also made them an excellent tool for the study of cellular physiology through their fusion to various proteins. These could be fluorescent proteins, yielding so-called chromobodies, to visually track live cell protein dynamics. Enzymatic degrader proteins have also been fused to nanobodies for targeted protein depletion, as have DNA-targeting moieties such as nuclease-dead CRISPR-associated protein 9 (dCas9) for locus-specific recruitment. Altogether, these provide a compelling set of tools for the tracing, marking, and manipulation of proteins in a native context.

The utility of nanobodies has also been augmented through the advent of modern conjugation techniques. Fluorophore-labeled binders are excellent tools for imaging, whereby nanobodies have proven superior for homogenous tissue staining as opposed to the bulkier, less penetrative antibodies. Nanobody size is also hugely advantageous in super resolution microscopy, providing miniscule linkage errors and thus enabling researchers to accurately resolve distances in the low-nanometer range. Beyond imaging, they have also gained much attention within biomedical applications; nanobodies have recently been used as tumor-penetrant drug conjugates, building blocks for advanced immunotherapeutic modalities, and targeting moieties for next-generation drug delivery systems.

In this review, we summarize the state-of-the-art of nanobody applications, detailing the advantages they bring to discovery and production systems, their unique biophysical traits, and their uses in studying intracellular dynamics. We also summarize their advantages in tissue-based and super resolution microscopy and finally their most recent applications as therapeutic modalities.

## DISCOVERY AND GENERATION OF NANOBODIES

### Synthetic and Immune Libraries

VHHs, or nanobodies, are *N*-terminal immunoglobulin (Ig) domains originally derived from the heavy chain-only antibody (hcAb) repertoire (1). These hcAb antibodies are a special class of antibodies found in camelids and sharks, devoid of the light chain. The resulting variable heavy (VHH, for hcAb) domain is therefore stable and monomeric, without needing to pair to a variable light (VL) domain. The VHH domain, thus like other Ig domains, comprises framework regions (FRs, 1–4) and hypervariable complementarity determining regions (CDRs, 1–3). Their ability to forsake the VL domain is accomplished by the evolutionary exchange of several surface-exposed hydrophobic residues for hydrophilic residues (2, 3). The FR2 region is a well-known hotspot for such mutations since this is the face of the Ig fold that usually interacts with a complementary VL domain. In addition, the VHH is known to have longer CDR3 regions, and a part of the CDR3 often folds back on the FR2, further stabilizing the monomeric fold (3, 4).

**Figure 1.**
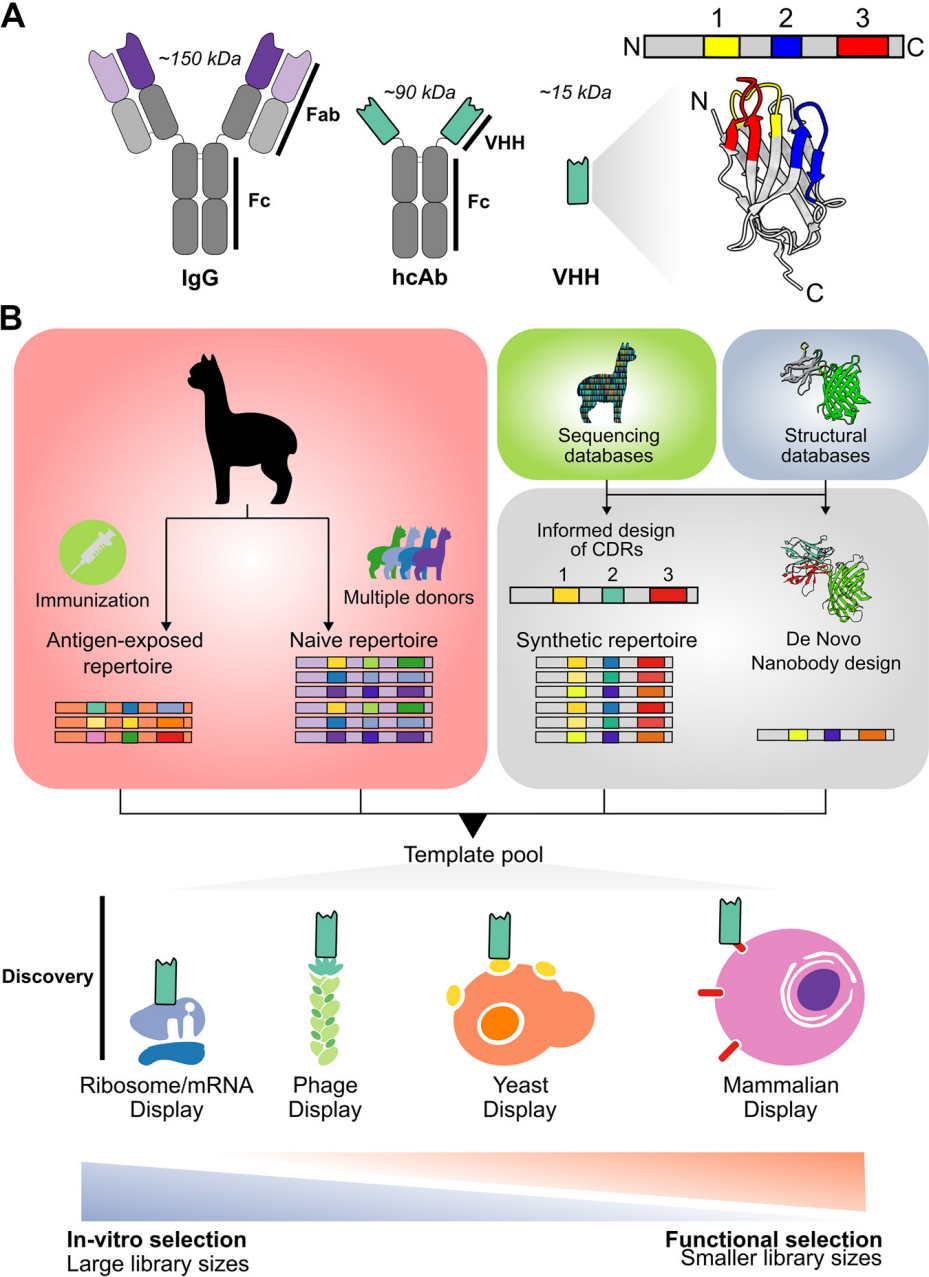
Design and discovery of nanobodies. *A* (*left*): schematic comparison of full-length IgGs and heavy chain-only antibodies (hcAbs). Light and heavy constant domains are colored in light and dark gray, respectively. *Right*: structural representation of an hcAb VHH domain or nanobody. CDR loops 1–3 are highlighted in yellow, blue, and red, respectively. *B*: summary of nanobody library generation and discovery methods. Antigen-exposed template pools can be obtained through camelid immunization, whereas naïve repertoires can be achieved by sampling multiple nonimmunized donors. Synthetic libraries can be designed through in silico rational design based on preexisting sequencing databases or by using structurally informed de novo protein design. The final template pool can then be used for discovery campaigns by methods such as ribosome, mRNA, phage, yeast, or mammalian display, with a general trade-off between library size and functional relevance. Structures depicted in this image are derived from the GBP-enhancer-GFP complex (PDB ID: 3K1K). CDR, complementarity determining regions; GBP, green fluorescent protein-binding protein; GFP, green fluorescent protein.

Ever since the hcAb repertoire was originally discovered in camelids and sharks (5–7), many successful attempts have been made to capture this diversity in the form of a library (Fig. 1) (6, 8, 9). Although antigen-naive repertoires have been sequenced and cloned into relevant display vectors, a notable approach is an immunized-donor-derived VHH library. This approach first starts with the immunization of healthy camelids. Then, a library can be obtained by cloning the repertoire of VHH-encoding sequences from antigen-exposed B cells, which is pre-enriched for antigen binding (10). A streamlined approach for the isolation of VHH sequences from a camelid donor involves RNA isolation, cDNA synthesis, and finally the amplification of the nanobody-encoding sequences. A two-step approach uses primers that bind the signal sequence and constant regions (CH2) of antibodies to first amplify nearly all antibodies of the donor, where the hcAb amplicons can be distinguished based on amplicon size. Subsequently, the hcAb amplicons can be functionalized in a second PCR to have the relevant enzyme sites for convenient cloning into a display vector (10). However, due to studies sequencing the VHH repertoires from alpacas, there have been significant optimizations in the primer design, allowing for a single-step amplification of VHH fragments (11). An immune library is therefore constructed with sequences amplified from an immunized donor, capturing the VHH diversity that encodes immunogen binding.

A simpler approach, albeit involving animal studies, leverages naturally present, recombined VHH sequences: the naive VHH library. In this approach, circulating PBMCs of several healthy camelid donors serve as a template for amplification of diverse recombined VHH alleles, which have natural diversity in not only the CDR loops but notably the framework regions as well, thereby capturing maximum functional diversity of the VHH pool (8). Synthetic libraries on the other hand are created by focusing amino acid diversity at the CDR loops. Two key aspects are important for most synthetic nanobody libraries: scaffold selection and diversity of the CDR loops. Although the interplay of CDR loops and scaffold together contribute to a functional binder, the scaffold is often chosen aimed at a key developability goal. For example, Moutel et al. (12) used a chloramphenicol acetyltransferase (CAT) reporter gene assay to select nanobodies from a pool capable of stably folding in the bacterial cytoplasm to discover the sdAbD10 scaffold. This scaffold aimed to improve the developability properties of the nanobodies derived from it, which would hopefully retain the intracellular folding capabilities of their parent. The Predator library used a fully human scaffold to generate a synthetic library of single-domain antibodies, with the objective of circumventing humanization efforts after binder discovery (13). Another unique scaffold based on the cAbBCII10 nanobody was chosen for its inherent stability, high production in *E.coli*, and ability to function in the absence of the conserved disulphide bond present in all nanobodies (14). This particular scaffold was selected for CDR grafting, an approach aiming to camelize known binders by simply swapping in CDRs from a known binder into the cAbBCII10 scaffold. There have also been efforts to use single scaffolds of well-studied nanobodies or antibody fragments, such as the sybody library, (15) based on the green fluorescent protein (GFP)-binding protein (GBP) nanobody scaffold and VH-B1a based on the Herceptin VH domain (16). In the former, the authors hoped to inherit the high stability and binding orientation of the GBP nanobody (17, 18), and in the latter authors aimed at recapitulating the favorable developability profile of the VH domain that had already seen clinical trials. In each of these libraries, the CDR loop diversity was entirely designed and is beyond the scope of this review.

Each library type offers complementary advantages and disadvantages. An antigen-exposed library is a pre-enriched, high-affinity pool of nanobodies and therefore makes it easier to obtain a binder of high affinity. However, since a large extent of selection already took place in the donor, the library sizes are smaller and less diverse (10). In addition, the processes that determine selection within the donor represent a black box and have not been studied extensively. Furthermore, the library is restricted to the immunized antigens, reducing its versatility for rapid binder discovery (12, 15). A naive library offers a middle ground; however, it requires several donors. Conversely, synthetic nanobody libraries are extremely versatile; they are not restricted to specific antigens and can also be used for toxic or harmful antigens. The sequences in a library can be entirely designed to allow for a diverse array of binding and biophysical properties. This results in a diverse yet defined pool, capable of binding with often lower affinities to a variety of antigens. However, lower affinity often needs to be addressed with an additional affinity maturation step, and their biophysical characteristics may not directly recapitulate the inherent stability of VHHs derived from natural sources (16).

### Discovery Strategies

Regardless of the type of library, the success of finding a potent binder that fits the specificity criteria of a project largely depends on the library size and the methods used to narrow down candidate binders. Nanobodies have notable advantages for several discovery strategies. The small, monomeric, stable protein provides biophysical advantages for reliable display in any context (19, 20). Since all antigen-binding capacity is captured in a single domain, it circumvents the need for large libraries commonly constructed to cover VH-VL combinations (21). Various display methods have been developed to sample the diversity of a library in a manner suited to the application of the nanobody. An ideal nanobody discovery system should allow an iterative filtering of the original pool, often performed in so-called rounds of enrichment. Of course, a robust genotype-phenotype link offers faster candidate selection and identification of nonredundant clones. On the other hand, an ideal screening system would allow purification-free high-throughput screening of selected candidates from the discovery pipeline and be as close to the final application of the nanobody (22).

To adequately sample the large library sizes commonly used for synthetic repertoires, phage display is a powerful technique that allows enrichment to take place in the scale of 10^13^ individual clones (23, 24). There are many proteins on the surface of an assembled phage, but most commonly, the nanobody library is cloned *N*-terminal to the low-copy PIII protein in a phagemid vector (25). Candidates obtained from a phagemid selection system are amenable to high-throughput screens such as ELISA either using phage-presented monoclonal nanobodies or as recombinantly produced histidine tag fusions (22). Some vectors such as the pHEN vector have an amber codon between the histidine affinity tag and the PIII DNA sequence, allowing expression of histidine tag fusions through the infection of a nonamber-suppressing strain, thereby avoiding subsequent cloning steps (26). In the current landscape, a bulk subcloning is often performed and diversity from the selection-display system is conveniently transferred to a screening system such as a pET vector-based expression system. Notably, ribosome display further samples a greater number of individuals from the population and has therefore been used in the context of synthetic libraries. The large sybody library, which is based on several scaffolds and diversity regions, led to theoretical library sizes of up to 10^22^. Binder affinities obtained by in vitro display largely depend on starting library size and diversity. The authors then used ribosome display to funnel the original large diversity to a phage display-based vector, highlighting the strengths of the in vitro discovery methods when used in conjunction (15).

Display is not restricted to the surface of bacteriophages, and efforts have been made to display antibody fragments on the surface of yeast and mammalian cells. Yeast display is a method that involves fusing the nanobody to the Aga2 protein, allowing display on the yeast surface (9). This allows binder selection and screening with fluorescence/magnetic-activated cell sorting (FACS/MACS) pipelines, benefitting from advantages of being a eukaryotic expression platform while maintaining single-cell high-density suspension cultures—often a prerequisite to sample large library sizes (27). Almost mirroring the yeast display system, mammalian display aims to further emphasize the context specificity of a eukaryotic system during the process of display and selection (28, 29). However, it has seen its use primarily in the discovery of full-length antibodies. Both these methods use FACS-based readouts, allowing purification-free high-throughput screening of several hundreds of clones. In addition, by manipulating antigen availability in the FACS assay, monovalent interactions can be preferred, and candidates can be screened directly for cell-based kinetics.

Although the surface of yeast is amenable to engineering and display, a unique form of discovery in the case of nanobodies is when it is performed in an intracellular context to discover so-called intrabodies. This method is called intracellular affinity capture (IAC) and is achieved by linking the nanobody-antigen binding event with the release of an auxotrophic phenotype, inspired by the yeast two-hybrid approach for protein-protein interactions (30). This leads to an affinity-dependent expression of a reporter gene, allowing binding clones to survive in selective medium. Stringency of selection can be modulated by challenging clones with increasing concentrations of the selectable marker. Similar to the yeast two-hybrid approach, a bacterial two-hybrid approach has also been developed for protein-protein interactions, allowing large library sizes (31). A parallel can be drawn to phenotypic screens of intrabodies that aided in the discovery of novel VHHs that inhibited the function of viral proteins (32). Importantly, each of these methods satisfy the primary criteria that define an ideal discovery (iterative enrichment and genotype-phenotype link) and screening method (high throughput, close to the final application of the nanobody).

Historically, most discovery techniques relied on display technologies that involve cloning the repertoire into a library; in the current landscape, there are several methods that are strikingly different. A notable approach was demonstrated by Fridy et al. (33), where they put a proteomic spin on nanobody discovery. Through the parallel assessment of mass spectrometry data of bound hcAbs and sequencing data from the same donor, they recapitulated the genotype-phenotype link and were able to discover several nanobodies for green fluorescent protein (GFP) and monomeric Cherry fluorescent protein (mCherry) across a broad affinity range. Recent studies have shown that nanobodies can be selected in silico and be entirely designed for specific epitopes on targets with the help of deep neural networks (34). Notably, nanobodies provide an additional advantage being short in sequence length and therefore require less computational resources for in silico modeling. A practical advantage of screening shorter sequences encoding the complete antigen-binding capacity is the lower costs for DNA synthesis.

## BIOPHYSICAL CHARACTERISTICS OF NANOBODIES

### Stability and Production

**Figure 2.**
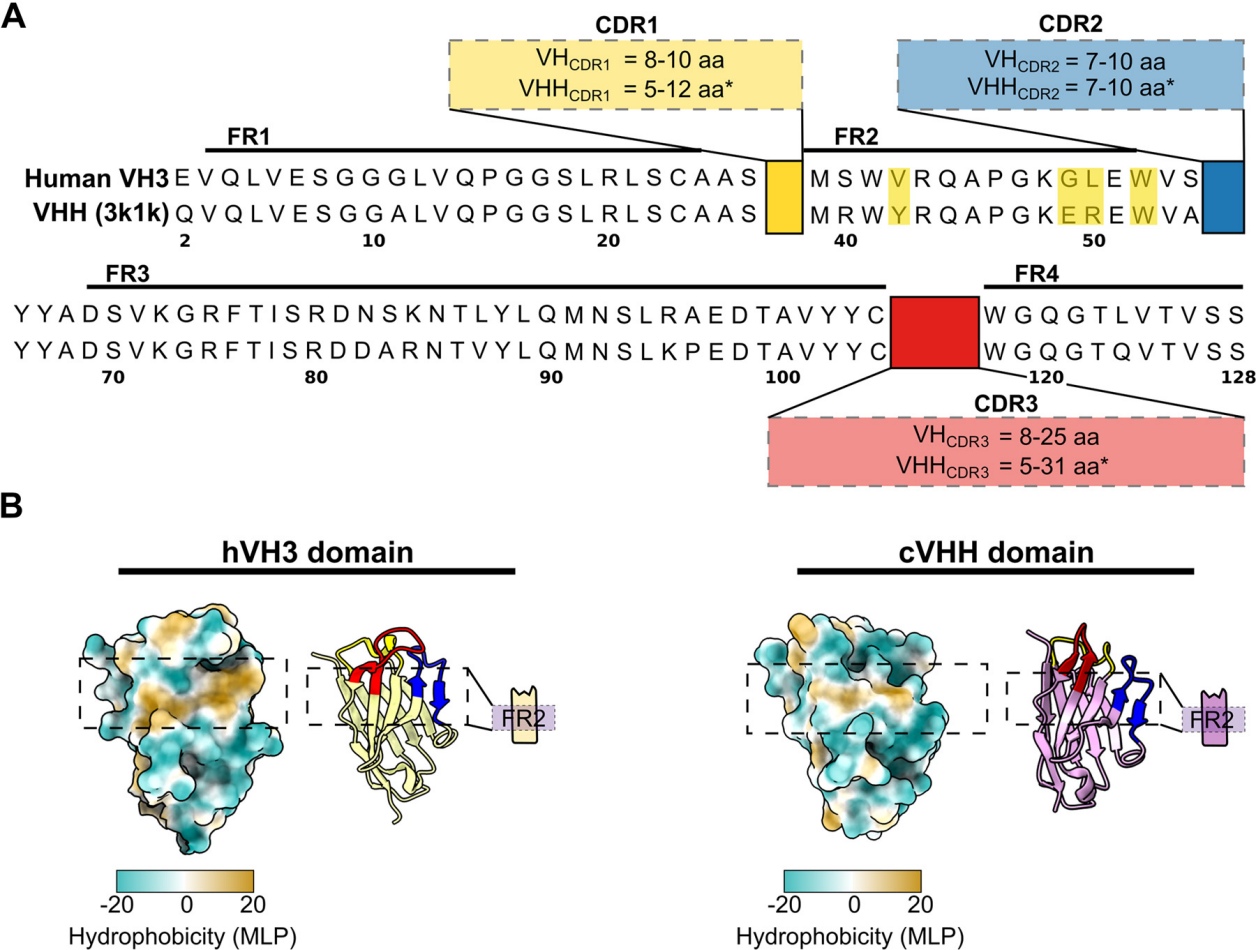
Nanobody sequence and structure. *A*: amino acid alignment (IMGT numbering) between a GFP-binding nanobody (VHH 3k1k) and a human VH3 domain. Hallmark mutations within framework 2 that incur nanobody monomericity are highlighted in yellow. *B*: structural depiction of VHH 3k1k and a VH3 (VH 1DEE), highlighting the comparatively reduced hydrophobicity of the former VL-binding interface on the VHH. Structures are colored by molecular lipophilicity potentials (mlp) with ChimeraX. *Cysteine residues are commonly found in the CDR1 or CDR2 of old-world and new-world camelid VHHs, respectively. These serve as conformational stabilizers by forming a disulfide bond with an additional cysteine in the CDR3. GFP, green fluorescent protein.

The discovery of nanobodies yielded a tool thus far unique in the arena of antibody-based binders. Unlike scFvs, which are contingent on an unnatural fusion between the VL and VH domains of a conventional IgG, nanobodies maintain full stability and binding capability as a single domain. This is largely due to the significantly higher surface hydrophilicity of the framework 2 (FR2) region of a VHH domain in comparison with a VH or a VL (3). Within an IgG, this hydrophobic patch is a key contributor to VL-VH pairing and thus makes these domains highly prone to aggregation if expressed individually (35–37). In contrast, VHHs derived from hcAbs are highly stable in solution since this FR2 interface is solvent exposed without the need to be shielded by an adjacent domain. These hallmark residues that confer FR2 hydrophilicity (and thus VHH monomericity) are summarized in Fig. 2, aligned against the germline sequence of the VH3 subfamily to which VHHs are most closely related (38). Another characteristic of nanobodies that aids their stability is the significantly longer average length of their CDR3 regions compared with conventional VH domains. Aside from potentially enabling access to cryptic epitopes (see *The Nanobody Binding Interface*), the long CDR3 regions of VHH domains are commonly observed to “fold back” onto the FR2 region, shielding additional hydrophobic residues that would otherwise be solvent-exposed (39).

This combination of solubility with structural simplicity renders nanobodies highly appealing compared with human-derived antibody fragments. Alongside their suitability for display and ease of discovery, another area of superiority is nanobodies’ exquisite production yields. They are routinely expressed in *E. coli* at highly scalable concentrations, owing to their simple folding, hydrophilicity, and frequent tolerance to reducing environments (12, 38, 40). In contrast, scFv expression in *E. coli* is often problematic, wherein interchain VL-VH mispairing, alongside their inherent hydrophobicity, results in aggregation and thus comparatively less functional protein expressed (35, 41, 42). Aggregation propensity can be alleviated by exporting proteins to the oxidizing periplasmic space, where disulphide bonds are allowed to form, and protein folding is aided by periplasmic chaperone proteins (43). However, since it is much smaller than the bacterial cytoplasm, export to the periplasmic compartment already incurs a cost on the maximum achievable protein yield (44). The ability of many nanobodies to fold in the reducing bacterial cytoplasm may also forsake the need for periplasmic export, potentially widening the gap of expression yields even further (12, 45).

Nanobodies, being naturally evolved domains, come with the added advantages of high thermal and conformational stability (35). They have been shown to denature at significantly higher temperatures than scFvs and have demonstrated a remarkable capability to refold into their proper three-dimensional (3-D) orientation after denaturation compared with human VH domains (44). This structural integrity is likely also conferred by the hallmark substitutions within the framework regions, as shown by reports of human VH domains that were drastically stabilized upon their camelization (artificially introducing FR2 hallmark mutations) (36). Vice versa, VHH humanization (mutating hallmark residues to resemble their human counterparts) has been shown to reduce thermostability and refolding capacity (46). Conrath et al. (47) demonstrated this by sequentially humanizing a trypanosome-binding nanobody, showing all variants to be more aggregation-prone, more easily denatured, and less capable of refolding. Interestingly, these variants also showed weaker antigen binding when the FR2 residue Tyr42 (IMGT numbering) was mutated, highlighting the importance of framework-CDR interactions that influence the binding properties of VHHs rather than solely biophysical stability. More recently, Kunz et al. (48) used a large-scale screen of ∼70 different nanobodies to assay them for stability under thermal and chemical denaturation. Their findings indicated that nanobodies with longer CDR3 loops were more robust under harsh conditions and refolded more efficiently than those with shorter ones, further highlighting the influence of CDR3-FR2 packing on protein stability. Another interesting finding was the lower aggregation propensities demonstrated by VHHs containing an additional cysteine pair between their CDR1 and CDR3 regions. These extra cysteines are most frequently present in dromedary-derived nanobodies and are reported to form disulphide bridges between CDR loops, thus compensating the potential cost to conformational stability incurred by prolonged CDR3 loops (49, 50).

Although fully human VH domains with comparable thermostability and solubility have been isolated, these are often heavily stabilized by their CDRs (51–53). As such, the diversification strategies for such VHs are constrained by these CDR-based stabilization motifs, which could reduce the maximum sequence diversity of discovery libraries based on such scaffolds compared with fully VHH-based ones. Ultimately, the choice between camelid and human-based scaffolds must be driven by the final application in mind. If the final goal is to generate binders for therapeutic use, then using human-like frameworks is likely necessary to avoid immunogenicity, likely at some cost to biophysical stability, sequence diversity, and scalability. On the other hand, camelid-based binders are supremely useful in primarily research-based applications. As a result of their natural stability, VHHs represent scalable, cost-effective molecular tools with profound amenability on both the chemical and genetic levels.

### The Nanobody-Binding Interface

A key property with respect to antibodies and antibody-derived fragments is affinity. Conventional antibodies create conventional paratopes with the combination of heavy chain- and light chain-derived CDR loops. This large surface offers a flatter, yet rich playground for amino acid diversity, largely responsible for the diverse types of antigens that antibodies can bind with high affinity. Nanobodies, lacking a light chain, have different strategies for high-affinity binding. First, single-domain antibodies are known to have longer CDR3 loops, extending the size of the offered paratope by simply increasing the length of the hypervariable region. Since some CDR3s have also been reported to fold back onto the framework regions, they further stabilize a conformation productive for binding. Nature plays a fine balancing act with the rigidity of such a protein; interloop disulphide bonds are more frequently observed (from CDR loops 1–3 and 2–3 for old and new world camelids, respectively), which play a crucial role in stabilizing nanobodies with extra long CDR3 loops. Another interesting phenomenon is highlighted by the ability of single-domain antibodies to bind to cognate targets with the help of residues in their framework regions (54).This is reflected in the unique binding interfaces nanobodies have been described to exhibit, such as concave, loop, and convex types, each classified by differing epitope sizes and accessibility. These properties have even been considered to generate large synthetic libraries (15).

Although some lines of research seem to suggest that single-domain antibodies bind cryptic epitopes or extend their long CDR3s into cavities of the antigen (4), a recent analysis of antigen-binder complexes shows that these interactions among single-domain antibodies and full-length antibodies may not be so different after all (54). Using solved crystal structures of nanobodies and antibodies interacting with their cognate antigens, it was found that the paratope of a nanobody tends to be smaller than that of an antibody. Interestingly, even though conventional antibody epitopes tend to be more linear, there was no other significant difference in the types of epitopes, nanobodies, and antibodies targeted. Parameters such as epitope size and amino acid composition seem to be very similar across nanobodies and antibodies. However, epitopes targeted by single-domain antibodies are slightly less accessible than those of conventional antibodies; even though the actual difference in accessibility seems small, this effect is still statistically significant. A key aspect to consider is the degree to which the discovery and screening method leads to discovery of specific binding modes or properties such as epitope accessibility. Nanobodies, most likely discovered by phage display or other recombinant technologies, tend to have unique properties not only because they are a group of diverse variable domains but also because display and recombinant technologies leverage strategies to correctly enrich specific binders. Proof of this concept dates as far back as 2010, when Kirchhofer et al. (55) discovered the GFP nanobodies. Since nanobodies in this case were sequenced after determining unique clonotypes, the authors narrowed down two main candidates that each bound their cognate target GFP to produce unique yet opposite effects. One increased GFP fluorescence (named enhancer) and the other decreased it (named minimizer), possibly capturing the conformational landscape of GFP during the biopanning and enrichment processes. Others have used prior knowledge to direct selection of binders that suited the goal of the biopanning campaign. G-protein-coupled receptors (GPCRs) are a family of receptors central to signaling in some of the most important processes (56). To crystallize them in the active conformation, multiple parameters would need to be optimized and each of these parameters would need to be compatible with every other optimization. By capturing the active conformation during the biopanning process, a binder dubbed Nb80 was enriched for binding the active conformation of the physiologically important β-2 adrenergic receptor (57). The conformation-selective nanobody provided an elegant solution to the larger goal, allowing structural studies of notoriously challenging proteins such as GPCRs. The same nanobody then was used to describe the ability of the internalized adrenergic receptor to contribute to signaling several minutes after agonist binding and internalization (58). Epidermal growth factor receptor (EGFR) is a physiologically critical receptor that is involved in cell growth and is notoriously misregulated in cancers. Along similar lines, conformation-sensitive nanobodies binding ligand-bound and ligand-free EGFR were discovered. Using these nanobodies, the authors described a novel intermediate “predimer” state of EGFR that was distinct from activated dimers (59). A significant advantage of nanobodies is their amenability to further optimization and selection of optimized binders satisfying a criterium. This is exemplified by a recent nanobody targeting the severe acute respiratory syndrome coronavirus 2 (SARS-CoV-2) spike protein. NbC5G2 was developed through an affinity-maturation strategy using a low-affinity clone C5 obtained from the original library. This low-affinity clone could compete with angiotensin-converting enzyme 2 (ACE2) receptor binding to the spike protein, suggesting neutralizing potential; however, its low affinity prevented it from being useful as a therapeutic. Once affinity matured, NbC5G2 showed broad-spectrum neutralization activity in pseudotyped virus assays (60).

Biologically, affinity is improved by somatic hypermutation, when a mutagenic enzyme [activation-induced cytidine deaminase (AID)] is driven to the immunoglobulin variable (IgV) gene locus in activated B-lymphocytes (61). A hidden improvement with binding strength lies in the avidity of the Fc-dimerized nanobody. Simple Fc-fusions have been shown to improve the functional affinity of binding by 2- to 10-fold, a property often taken for granted with full-length antibodies (62, 63). An alternative approach is a tandem nanobody fusion, which also forms a bivalent binding unit and therefore drives avid binding. This approach can take information from epitope binning experiments to construct so-called “super binders,” which are multiparatopic fusions targeting the same protein, but different epitopes on its surface (64). These tandem fusions can achieve even greater specificities and avidities, improving their use for certain applications such as protein purification.

## NANOBODIES AS MOLECULAR TOOLS

### Nanobody-Based Genetic Fusions

Over the past decades, the study of proteins has gotten progressively more detailed, necessitating the use of highly sensitive and specific tools to distinguish finer differences. Antibodies have been some of the most well-studied groups of proteins due to their singular remarkable property, their ability to bind to and recognize their cognate antigen. They can be used as detection reagents in Western blots, as staining reagents in FACS and imaging, and can be used to pull down native complexes with unparalleled specificity for native proteins. Antibodies are indispensable to some of the most important developments and discoveries in the field of biology. In recent years, the focus has developed further into the functional dynamics of proteins and now require live cell methodologies. With the advent of CRISPR gene editing and several improvements in genetic engineering, introducing perturbations and modifications of near-native proteins has been widely successful, allowing their study at endogenous expression levels. Equally important, and much like their larger counterparts, nanobodies provide a toolbox for the study of intracellular proteins in near-native contexts as well. However, unlike full-length antibodies, nanobodies provide an additional advantage: a modular platform for the intracellular perturbation, modification, and study of protein function in the live cell context. Antibody derivatives active within cells are often referred to as intrabodies (65).

**Figure 3.**
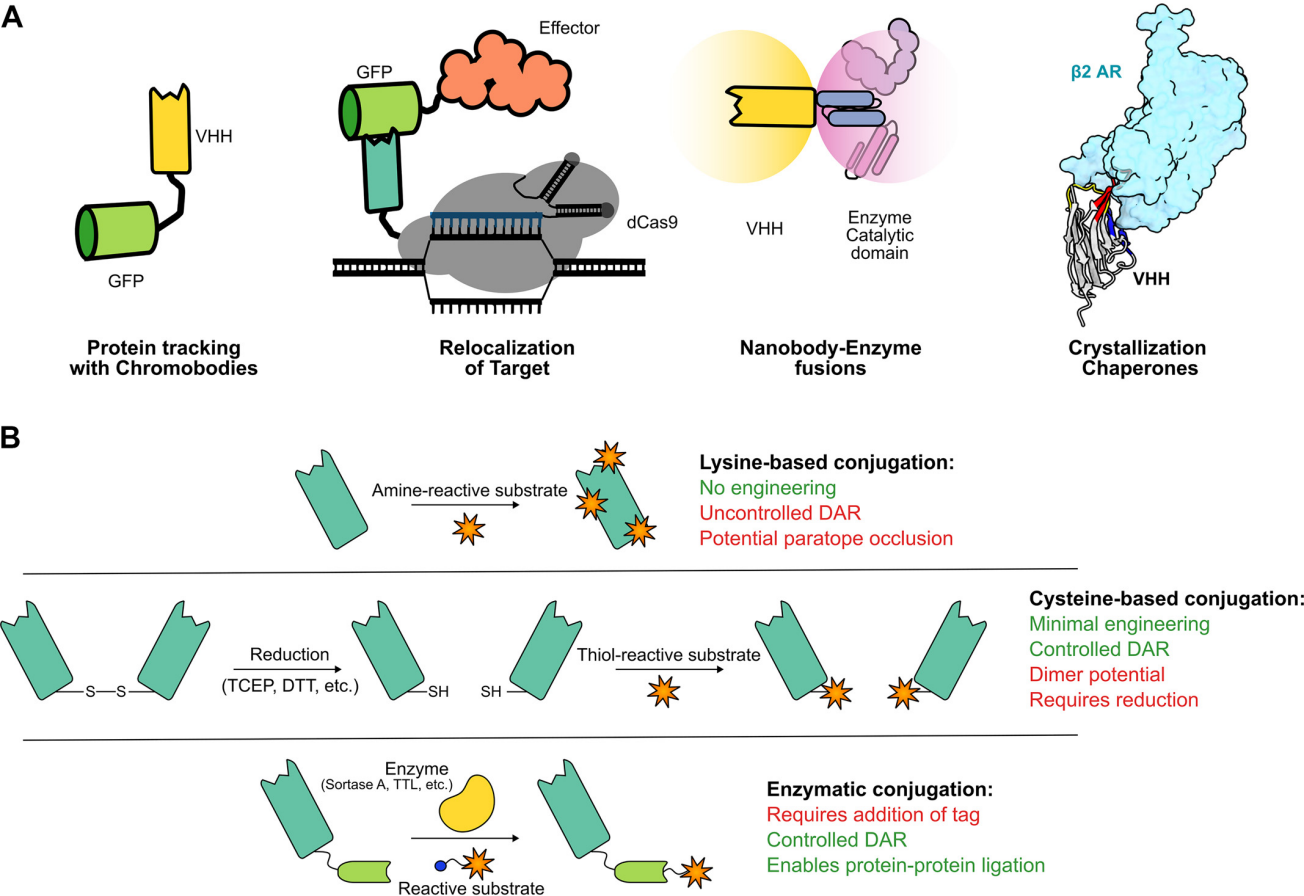
Modes of nanobody functionalization. *A*: summary of nanobody applications for molecular study. This includes nanobody fusions to fluorescent proteins (chromobodies), recruitment of effectors to genetic loci, protein manipulation by targeted enzyme catalysis, and crystallization stabilization for protein structure determination. *B*: summary of conjugation methods commonly used for nanobodies including stochastic lysine conjugation (*top*), engineered cysteine incorporation (*middle*), and chemoenzymatic ligation (*bottom*).

Since nanobodies are derived from antibody variable heavy sequences, they share many properties with the conserved Ig fold, allowing a stable, well-expressed, and well-folded domain capable of binding specific structures in the cell. Leveraging their high affinity to their target, the nanobody can be used for relocalization, recruitment of enzymatic machinery, and simply tracking the protein of interest in a live cell (Fig. 3*A*). By the genetic fusion of the nanobody as a binding unit to a fluorescent protein for visualization, the dynamics of proteins can be observed inside living cells (18). These so-called chromobodies have been used for the live cell imaging of several intracellular antigens, providing a powerful alternative to genetic fusions with fluorescent proteins. This was elegantly demonstrated using proliferating cell nuclear antigen (PCNA), a protein known to change its cellular localization pattern through the cell cycle stages, particularly during the S phase (66). Fusion of a nanobody targeting PCNA to a fluorescent protein allowed for the classification of cell cycle stages and furthermore into individual S phase substages. In other applications, the dynamics proteins of interest can be tracked and potentially related to function. For example, a single high-affinity nanobody allowed the study of HIV in living cells (67). This study clearly highlights the advantages of using nanobodies for the study of a dynamic process such as virion assembly, where timing, localization, and pattern are critical to studying protein function.

Nanobodies can also be used for the relocalization of proteins to specific subcellular compartments of the cell. When transfected, a nanobody fusion with a localization signal or protein that assembles in a defined structure can traffic its target into the correct subcellular compartment as exemplified by the fluorescent three-hybrid (F3H) assay (68). In this assay, the GFP-binding nanobody GBP1 (17, 18) is fused to a lacI repressor protein followed by a nuclear localization signal (NLS). When expressed in engineered cells that contain an array of the lacO operator, the nanobody is recruited to the genomic locus containing the lacO array, and with it anchoring any GFP-tagged protein of interest that is cotransfected. This assay thus turns proteins of interest without any clear localization pattern into a defined cellular structure, which is then amenable to protein-protein interaction studies with red-labeled interactors. Furthermore, it can be an imaging-based screening system for intracellular interactions, which may be relevant for drug discovery.

Relocalization to genomic loci is especially interesting when nanobodies are fused to a guided dCas9 protein, allowing targeting of virtually any DNA sequence in live cells. A nanobody-dCas9 fusion recruits the target protein to the specific genomic locus, where the target protein can perform its function. This effect is exemplified by the GBP-dCas9 fusion, which can recruit GFP-tagged epigenetic modifiers such as ten eleven translocation 1 (TET1) and DNA methyl transferase 3 A (DNMT3A) catalytic domains (17, 69). This approach could have distinct advantages over direct catalytic domain fusions with the Cas9 protein by providing modularity, and by creating structural flexibility and dynamic local concentration of effectors.

In a similar vein, nanobodies have also been used to study proteins such as methyl CpG-binding protein 2 (MeCP2). MeCP2 is part of the methyl CpG-binding domain (MBD) protein family, which is frequently mutated in Rett syndrome, a neurological disorder (70). In healthy cells, MeCP2 accumulates at constitutive heterochromatin, performing its function as a transcriptional regulator. Although mutations in its MBD domain contribute to chromatin binding, several mutations affect heterochromatic clustering (71). Indeed, some mutations may affect both functions and it is difficult to determine causality. Leveraging the high affinity and specificities of nanobodies, the authors drove recruitment of GFP-tagged MeCP2 mutants to pericentric heterochromatin, allowing the study of mutational effects on heterochromatin clustering (72). Essentially, it revealed phenotypes of MeCP2 mutants independent of chromatin binding.

A unique case in which nanobodies have a distinct advantage is protein transfection, a rather understudied technique. Cell-penetrating peptides (CPPs) have existed and evolved naturally through the evolutionary arms race of host-virus interactions. When fused to a small, monomeric nanobody, they permit the access of intracellular components to nanobody binding in live cells (73). Once inside, the nanobody cannot only bind its target but is also capable of relocalizing it to cellular compartments such as the nucleolus. In addition, the nanobody is also capable of cotransporting its target, opening up possibilities for therapeutic delivery of proteins. Leveraging their amenability for recombinant expression, cell-penetrating nanobodies have seen use for live cell microscopy using super resolution-stimulated emission depletion (STED)-compatible conjugates (74). This allowed super resolution-stimulated emission depletion (STED) microscopy of live cells, preserving ultrastructure and reducing linkage error due to the use of nanobodies targeting GFP, mCherry, Lamin, and PCNA as primary staining reagents. This presents a promising alternative strategy, since live cell super-resolution microscopy was otherwise restricted to genetic fusions of target proteins, which may perturb native function of the target.

### Nanobody-Enzyme Fusion Proteins

Several multidomain enzymes separate recruitment and catalytic domains. Nanobodies can serve as binding units when fused to enzymes, retargeting enzymatic activity to alternative substrates. This forms the basis of enzyme-catalyzed proximity labeling methods. A simple example of this would be a Bio-ID assay, where a single-domain antibody is fused to the promiscuous biotin ligase enzyme BirA (75). Since the nanobody specifically targets the antigen when expressed in a cell, it can direct enzymatic biotin ligation to not only the antigen but also nearby interactors, allowing interactome analysis via a streptavidin pull-down. Thus, transient interactions that could be temporally regulated or are too weak for regular coimmunoprecipation can still be captured by the high-affinity strepavidin-biotin interaction. In addition, due to the strong interaction, once bound the matrix can be washed under stringent conditions, preventing contaminant carryover. The use of nanobodies as targeting modalities can therefore be extended further to other enzymes. Native modifications of proteins, also known as posttranslational modifications (PTMs), play crucial roles in their function, localization, and activity. It is therefore possible to fuse PTM catalytic domains to nanobodies, resulting in PTMs of their cognate antigens and nearby interactors. In some situations, modulating the PTM state of a target has proven to have a direct effect on its function. A key example of this was described recently by Kuo et al. (76), where the authors directed thioesterase activity to palmitoylated proteins. Palmitoylation is a lipid-based PTM that is emerging as a therapeutic target. Here, the Ca(v)1.2 voltage-sensitive calcium channel was chosen, since palmitoylated Ca(v)1.2 was associated with heart arrhythmias. Using a novel nanobody-thioesterase fusion, they directed specific depalmitoylation of the calcium channel and showed a reduction of early-after depolarization (EAD) frequency in an in vitro iPSC-cardiomyocycte model. Whether or not such a system will ever reach clinical therapy could be argued; however, this clearly highlights the utility of nanobodies to study protein function and the effects of PTMs in live cells. Similar studies have also been published that explored targeted dephosophorylation (77), targeted glycosylation (78), and conversely deglycosylation (79), all using nanobodies fused to catalytic domains of enzymes.

The use of enzyme-nanobody fusions has not been restricted to intracellular pathways. A novel αPD-L1 [programmed death ligand 1 (PD-L1)] nanobody was fused to a sialidase, an enzyme that catalyzes the removal of the sialic acid modification (80). Sialic acid is a negatively charged glycan, which often modifies cell surface proteins regulating critical processes such as adhesion, migration, and immune recognition. The latter of which is especially important when considering extracellular therapeutics, where sialic acid modifications mask cell surface antigens and may deregulate clearance of sick cells (81). This novel fusion provided a native solution to cancer therapy by repolarizing tumor-associated macrophages rather than directly depleting cancer cells.

Taking this concept further, several researchers have explored nanobody E3 ubiquitin ligase fusions (82, 83). E3 ubiquitin ligases modify proteins with ubiquitin, possibly one of the most well-studied PTMs. It is one that plays a key role in cellular regulation, whereby ubiquitinated proteins are often marked for proteasomal degradation. Using the GFP-GBP system (17, 18), several E3 ligases were screened for protein depletion by replacing their cognate substrate-binding domains with GBP1. This led to the identification of the Lnx1 RING domain as a promiscuous E3 ligase that could be redirected to degrade novel targets (84). Similarly, fusing catalytic domains from E3 ligases such as NSlimb and SPOP led to the targeted degradation of the cognate antigen in cytoplasmic and nuclear compartments, respectively (85). Importantly, this strategy can be extended to other model organisms with their evolutionary homologues, suggesting a broad applicability of the principle. More recently, the same strategy was extended to a proteome-wide screen to discover more proximity dependent E3 ligases (86). In parallel, the study also discovered several stabilizer modules that, instead of degrading the cognate antigen, increase its abundance. Taken together, nanobody-E3 ligase fusions or degraders leverage the high specificity and affinity of the nanobody to redirect enzymatic activity to novel targets, allowing researchers to explore phenomena such as driver oncogenes and haploinsufficiency.

### Nanobodies in Structural Biology

Nanobodies, like their full-length counterparts, can be used for purification. Some notable examples include tag-binding nanobodies targeting GFP (17, 18) and short peptides such as the ALFA (87) and Spot tag (88). Interestingly, nanobodies that bind purification contaminants have also been discovered, which solve the inverse problem of protein purification by getting rid of an abundant contaminant (89). Often touted for their stability, the compact structure of nanobodies offer advantages over conventional antibodies in their recalcitrance to harsh buffer conditions, increased thermostability, and longer shelf life. Ultimately, a nanobody used for purification can also be used for coimmunoprecipitation, allowing protein-protein interaction studies. Due to the modular nature of nanobodies, they can also be combined in tandem combinations to create multiparatopic constructs (64). These tandem fusions notably increased pull-down efficiency when demonstrated with the GFP antigen. Such tandem fusions have also improved staining reagents such as the Spot-nanobody (88).

Although subtle, the 2012 Nobel prize-winning studies of G-protein-coupled receptors also featured a nanobody, this time as a crystallization chaperone (56, 90). The preparation of diffraction quality crystals is a major impediment to structural studies of proteins. Often, crystallization trials involve testing many parameters such as ions, additives, and detergents in a high-throughput manner (91). An additional variable that often improves crystal quality is the addition of crystallization chaperones (92). These improve chances of creating well-ordered crystals by reducing conformational heterogeneity and by masking regions on the target that have low propensities to form productive crystal contacts. Historically, this role was attributed to interacting proteins and remains an effective strategy aiding crystallization (93). However, not all proteins have known binding partners, and the success of the crystallization trials ultimately depends on the affinity, chemical stability, and solubility of the binding partner, all of which are elegantly tackled with nanobodies. The first application of antibody-derived chaperones for membrane proteins was an Fv fragment (94), and subsequently until the discovery of nanobodies, Fab fragments were used due to their ease of production from monoclonal hybridomas following papain digestion (95). The conserved Ig fold additionally provides a unique advantage specific to antibody-derived fragments by assisting crystal nucleation with exposed beta sheets (96). One aspect that nanobodies have an explicit advantage in is their size. High-resolution protein nuclear magnetic resonance (NMR) is a structural determination method compatible with solution studies, capturing the lively dynamics of proteins. The size of the protein envelope accessible by protein NMR is challenging for proteins greater than 40 kDa, disqualifying complete IgGs and Fab fragments for this method. In contrast, nanobodies that are 10 times smaller in size are well suited and also provide additional advantages such as production in heterologous systems (97–100).

Cryogenic electron microscopy (CryoEM) is another method of structural determination that has seen landmark improvements in recent years. Although this method excels at describing protein structures, there is a caveat. Structure resolution struggles with smaller particles, and ironically, small proteins are abundant in nature, necessitating the use of fiducial marks for determination of their structures by cryoEM (101). Serving as protein modules that hold the complex in place, fiducial marks improve not only particle alignment, but also symmetry. Finally, they increase particle size, improving signal-to-noise ratios, thus allowing robust structure reconstruction. Leveraging their ease of production and affinity, nanobodies can be used to purify whole complexes from native lysates. Nanobodies, due to their small size, do not improve particle size by much. However, several modifications that improve its size such as the NabFab (102), Megabody (103), and LegoBody (104) have been developed, paving the way for nanobodies in cryoEM. BRIL-based technologies provide universal fiducial marks for cryoEM; however, they involve engineering the alpha helical BRIL sequence (an engineered apocytochrome b562) into target proteins, possibly risking the native structure (105). Recently, the Gembody (Gb) strategy was developed, possibly hammering two nails with one strike (106). By mutating several residues in the framework 1 and 4 regions of the nanobody, a kinetically assisted, symmetric outward facing orientation of nanobodies can be achieved. This concept is developed further into the di-Gembody (diGb), which introduces an engineered disulphide bond to enable controlled C-terminally disulphide-linked nanobodies. This novel engineering strategy could yield not only homo-diGbs but also bispecific hetero-diGbs, simultaneously increasing fiducial mass and allowing structure determination of two targets under the same campaign. A unique discovery that the authors emphasized was that due to the kinetically trapped, symmetric interface, nanobodies of micromolar affinities served as fiducial markers for reliable structure determination. Considering the ease of nanobody discovery and their amenability to high-throughput protein production, the engineered Gb scaffold permits structure determination regardless of affinity and massively extends the horizon for nanobodies in structural studies.

### Chemical Conjugation of Nanobodies

Although nanobodies shine in the realm of genetic fusions, they have also seen wide use as chemical conjugation partners. Over the past two decades, the array of protein conjugation techniques has expanded massively: beyond stochastic labeling methods such as NHS-based chemistry, a variety of strategies now exist to functionalize proteins in a much more specific manner (Fig. 3*B*). This is especially exciting in the context of antibody engineering, with the modern conjugation toolbox paving the way for the development of novel antibody-fluorophore conjugates (AFCs), antibody-drug conjugates (ADCs), and antibody-protein conjugates among others. However, the size and complexity of full-length antibodies can present a challenge in achieving favorable stoichiometries in chemical conjugations, a particular issue when functionalizing with bulkier substrates. In this sense, nanobodies are often preferred when generating more complicated chemical fusions, such as DNA conjugates or protein-protein ligations.

Site specificity is usually achieved by chemoenzymatic labeling of an engineered enzyme recognition motif. One popular example is modification through sortase A, a transpeptidase originating from *Staphylococcus aureus* that specifically recognizes the LPXTG motif, otherwise known as the SorTag. Sortase-based labeling is by now well-established and has been used to efficiently modify nanobodies with fluorophores (88), toxins (107), oligonucleotides (108), and protein domains (109, 110). Witte et al. (110) showcased the amenability nanobodies within SorTag platform by generating fully functional, C-C fused bispecific binders with impressive conversion rates. TubTag technology, developed in 2015, is a platform that also leverages a terminal enzymatic recognition site (111). The VDSVEGEGEEEGEE (TubTag) sequence is recognized by tubulin tyrosine ligase (TTL) to install a click-reactive tyrosine derivative, enabling flexible downstream conjugation. TubTagging was also used to generate C-C-fused nanobodies at high (50%–60%) efficiency, providing another viable method to produce unimpaired, outward-facing bivalent/bispecific binders. Schwach et al. introduced another application of the TubTag system by efficiently conjugating a GFP-binding nanobody to C-terminal DNA (or PNA) sequences for reversible staining in confocal microscopy (112). Other less-explored enzymes for site-specific nanobody labeling include microbial transglutaminase (113), formylglycine-generating enzyme (114), and butelase 1 (109).

An additional benefit of nanobodies is the ability to use cysteines as a nearly site-specific handle. Most nanobodies have only two cysteines that are buried (115), whereas full-length IgGs contain eight exposed cysteines, which form interchain disulphides. Although maleimide-based labeling of these exposed residues is an industry standard for IgG conjugation, the method is still fundamentally stochastic and thus difficult to control. Conversely, exposed cysteines can be engineered into nanobodies to provide precisely localized conjugation sites. In this sense, C-terminal cysteine introduction has proven a viable means to produce well-defined nanobody conjugates (116–119). Zavoiura et al. (120) applied this principle in a therapeutic context, using engineered C-terminal cysteines to site specifically label anti-EGFR nanobodies with siRNAs. The labeling method was expanded upon by Pleiner et al. (115) in 2015, who identified six positions within a nuclear pore-targeting nanobody to introduce accessible cysteines. All six positional variants could be efficiently purified and conjugated, using a gentle reduction procedure to leave the intrachain disulphide bond intact. In immunofluorescence experiments, singly labeled cysteine conjugates drastically outperformed stochastic lysine-based labeling, which resulted in high background, likely due to hydrophobic interfaces and CDR occlusion.

### Nanobodies in Imaging

**Figure 4.**
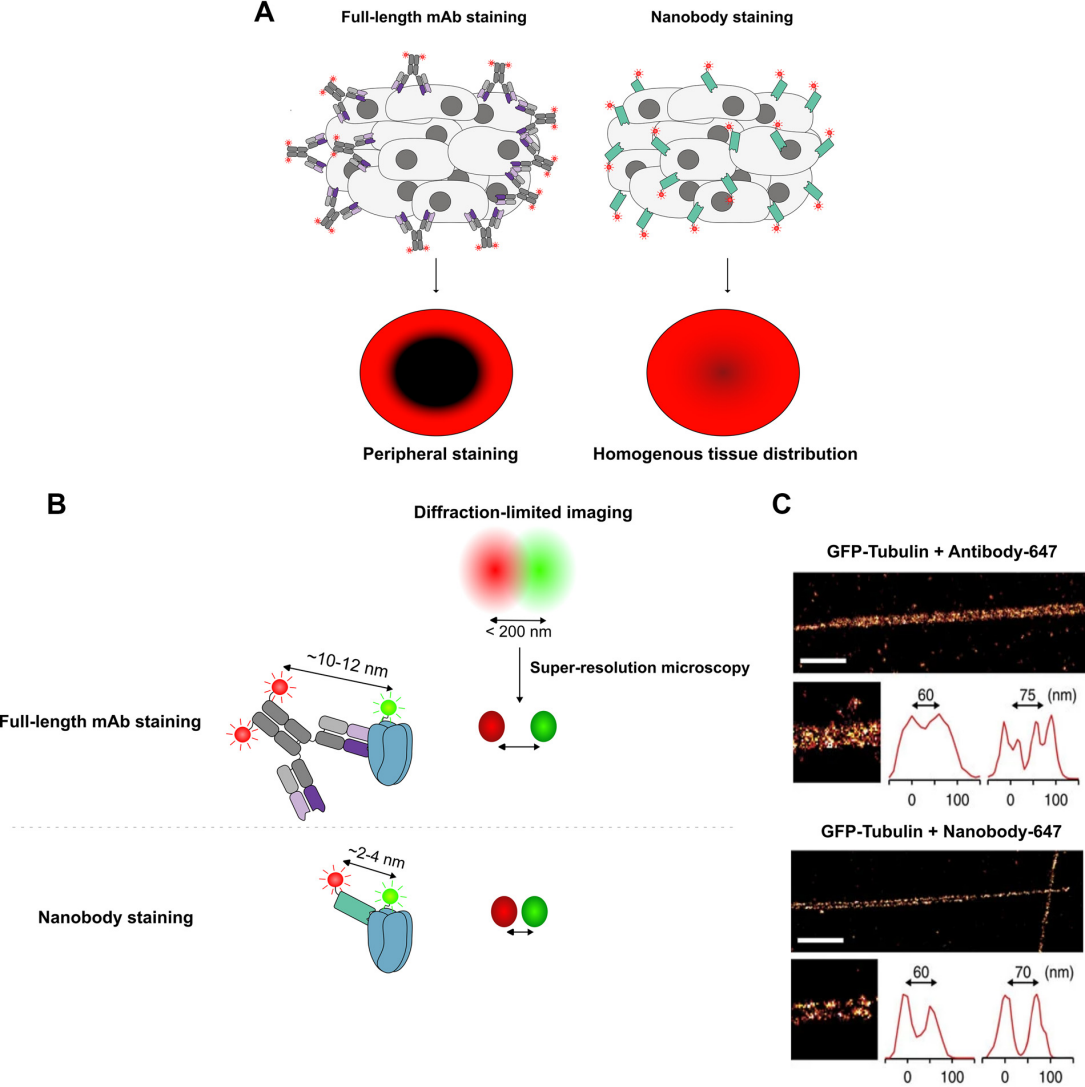
Advantages of nanobodies in imaging. *A*: schematic representation of tissue staining using full-length antibodies (*left*) and nanobodies (*right*). Due to their bulky nature, full-length antibodies suffer from restricted diffusion and can thus result in artifactual staining limited to the sample periphery. Conversely, smaller nanobodies can more freely diffuse inwards, resulting in homogenous staining. *B*: schematic representation of linkage errors introduced by full-length antibodies and nanobodies. Superresolution microscopy enables the distinction of diffraction-limited distances (<200 nm). At such distances, the minimal linkage error of nanobodies (∼2–4 nm) provides more precise signal localization relative to full-length antibodies. *C*: representative examples of linkage error effects in STORM imaging. GFP-tubulin stained by a full-length AF647-conjugated antibody results in suboptimal resolution between closely spaced microtubules. Conversely, due to minimal linkage error, nanobody conjugates can much more efficiently distinguish microtubules within a single bundle. Imaging data were sourced from Mikhaylova et al. (124) with author permission. Scale bar: 1 µm. GFP, green fluorescent protein; STORM, stochastic optical reconstruction microscopy.

Imaging systems can benefit tremendously from nanobodies and their advantages (Fig. 4). Aside from their ease of discovery, production, and functionalization, nanobodies provide a unique advantage for imaging due to their small size. Even though single-domain antibodies can often withstand harsh conditions such as high-salt buffers and denaturing agents, maintaining the quality of the sample used in the experiment is of utmost importance. This effect is exemplified by the recent technique nanobody-assisted tissue immunostaining for volumetric EM (NATIVE) (121). The authors aimed to combine fluorescence imaging and electron microscopy for the large-scale 3-D reconstruction of tissue sections at high resolution. Due to its small size, a single-domain antibody (the GFPenh nanobody) could penetrate weakly permeabilized aldehyde-fixed tissue with good staining intensity up to 100 μm in 500-μm thick brain sections. In contrast, they showed that an anti-GFP monoclonal antibody only stained the surface of the sample. Thus, by using nanobodies and avoiding harsh permeabilizing agents, they were able to preserve the ultrastructure of their samples. They also leveraged the better penetrance of fluorescently tagged nanobodies to image the same sample in the fluorescence and EM regimen, allowing fluorescence-guided EM reconstruction without damaging membranous processes. This is especially important for the study of neurons and brain tissue sections, where much of the functional relevance of these samples is driven by fine morphological differences in the very same membrane-associated events such as synaptic processes, mitochondria, and intracellular assemblies of proteins.

In 2011, the GFP-binding nanobody GBP (17, 18) was used to boost GFP signal while imaging the process of cortical abscission (122). This marked the first use of a nanobody in super-resolution microscopy (SRM), also known as nanoscopy: a category of imaging techniques that overcome the diffraction limit of ∼200 nm in the *xy* plane. Examples of these methods include structured illumination microscopy (SIM), photoactivated localization microscopy (PALM), stochastic optical reconstruction microscopy (STORM), and stimulated emission depletion microscopy (STED). Although the machinations of these techniques are beyond the scope of this review, one commonality between them is the ability to localize fluorescent signals down to a single-dye molecule through the manipulation of other fluorophores’ excitation states (123). At such resolutions, which can be as fine as ∼10 nm, the size of the imaging probe itself becomes relevant in defining molecular localizations. Fluorophore-labeled antibodies have long been the workhorses of fluorescent imaging and have a length of 10–12 nm on average. As such, in nanoscopy techniques, full-length antibodies cause a resolvable offset between the imaged fluorophore and the target structure,- a phenomenon known as the linkage error. This issue is only exacerbated when staining with an unlabeled primary antibody followed by a fluorescent secondary. Nanobodies present a compelling solution to this problem, owing to their miniscule length of just 2–4 nm. Mikhaylova et al. (124) exemplified this in a study directly comparing nanobodies and full-length antibodies for the imaging of neuronal microtubules via STORM. These structures are roughly 25 nm in diameter and are spaced in a lattice configuration at distances of ∼20–70 nm apart. Remarkably, the authors show that their in-house-generated VHHs reduced displacement to just ∼2.5 nm when measuring microtubule diameter, compared with ∼12.5 nm when using directly labeled primary antibodies. Furthermore, they were able to distinctly resolve ∼50% of microtubule structures spaced 25 nm apart, a 10-fold increase in resolution efficiency when compared with conventional primary/secondary antibody staining. Akter et al. (125) also demonstrated the superiority of nanobodies in resolving the subcellular architecture of neuronal synapses through STED. However, in this study, the authors also showed that antibody-based staining showed equivalent resolution capability with an alternate, more permeable fixation method. Since the target structures were ∼150 nm apart, this observation highlights that the difference in linkage error between antibodies and nanobodies chiefly comes into play when measuring much smaller distances. However, it also demonstrates the benefits of nanobodies’ other unique attributes, such as penetration and reduced steric hindrance, in nanoscopy.

The main bottleneck of nanobody-based SRM is a dearth of specific binders in comparison with antibodies, which have enjoyed a decades-long head start in terms of broad discovery campaigns. However, tag-binding nanobodies such as GBP (17, 18) present a reliable, if slightly artificial, shortcut to maximizing the capabilities of nanoscopy at high-throughput scales. This was showcased by Ries et al. (126), who detected tubulin-YFP with GBP-AF647 to achieve exquisite resolution of its diameter. Although a GFP-targeting antibody visualized a diameter of 42.7 nm, the GBP nanobody resolved it at 26.9 nm, accurately reflecting tubulin’s true diameter of ∼25 nm (126). This prompted the authors to generate an array of 20 GFP fusion proteins in yeast and image them in high throughput using the same nanobody. Due to the minimal linkage error, labeling efficiency, and accessibility of nanobodies, the authors could visualize distinct nanoscale organizations of these proteins, such as that of Cdc11 during yeast cell division. In 2021, Schneider et al. (74) used tag-specific binders to capitalize on other advantages of nanobodies in SRM. Here, GBP was simultaneously conjugated to a fluorophore, a photostabilizer, and a cell-penetrating peptide (CPP). Using this triple conjugate, the authors were able to achieve transfection-free super-resolution imaging of intracellular GFP-PCNA through STED. Due to the presence of a photostabilizer, this configuration was also compatible with live cell imaging, allowing resolution of single-replication foci over 30 STED imaging rounds. This setup was also shown to be translatable to mCherry-tagged proteins and untagged endogenous proteins. Taken together, these experiments are an elegant showcase of nanobodies’ ease of functionalization, penetration capabilities, and excellent compatibility with fine STED-based imaging. Another tag-based nanobody was developed in 2019 with the specific goal of flexible imaging in mind, raised against the so-called ALFA tag (87). This is a short helical sequence with excellent biophysical properties, which can thus be incorporated into a target protein sequence with minimal perturbations. Its cognate nanobody (NbALFA) could bind at ultrahigh affinities regardless of tag placement at either terminus or even between protein domains. NbALFA was also shown to produce linkage errors as low as >3 nm, setting the stage for its use in SRM.

A further nanoscopy method for which nanobodies have recently found use is DNA-PAINT. This is a technique that relies on the hybridization of dye-labeled imager DNA strands to complimentary docking strands linked to a target of interest, usually through an antibody. Due to the transient binding (blinking) of the imager strand, it is possible to achieve resolutions of >5 nm, provided that enough images are acquired for algorithmic spot localization (127). Minimizing linkage error is of course imperative at such dimensions, and hence nanobodies have become an ideal tool for labeling proteins in DNA-PAINT (128). Resolution enhancement by sequential imaging (RESI), a pioneering DNA-PAINT method allowing the resolution of single molecules down to the Ångström level, accentuated the utility of nanobodies in the next generation of imaging (129). In this landmark study, a GFP-binding nanobody was used to label transfected CD20-GFP and measure the distances between monomers with and without CD20 antibody engagement, leading to a precise quantification of monomer and dimer species on a single-molecule level. This method can also resolve antigen-binder complexes, as shown by a recent follow-up study investigating the effects of two anti-CD20 antibodies on the antigen’s nanoscale organization (130). This was achieved by the simultaneous nanobody-mediated labeling of a C-terminal ALFA tag on bound antibody and an intracellular GFP on the antigen. As a result, the authors could achieve an effective resolution of 2–5 nm, restricted only by the minimal linkage error introduced by the nanobodies. This enabled them to, on a single-molecule level, distinguish the unique clustering patterns of type I and type II CD20 antibodies in complex with the antigen, visualizing the oligomeric complement-engaging C1q platforms induced by type I antibodies. In a broad sense, RESI represents a tremendous leap toward visualizing and understanding therapeutically relevant events at low-nanometer distances, and in that vein has virtually maximized the potential of nanobodies in superresolution microscopy.

## NANOBODIES IN BIOMEDICINE

### Nanobody-Based Conjugates

**Figure 5.**
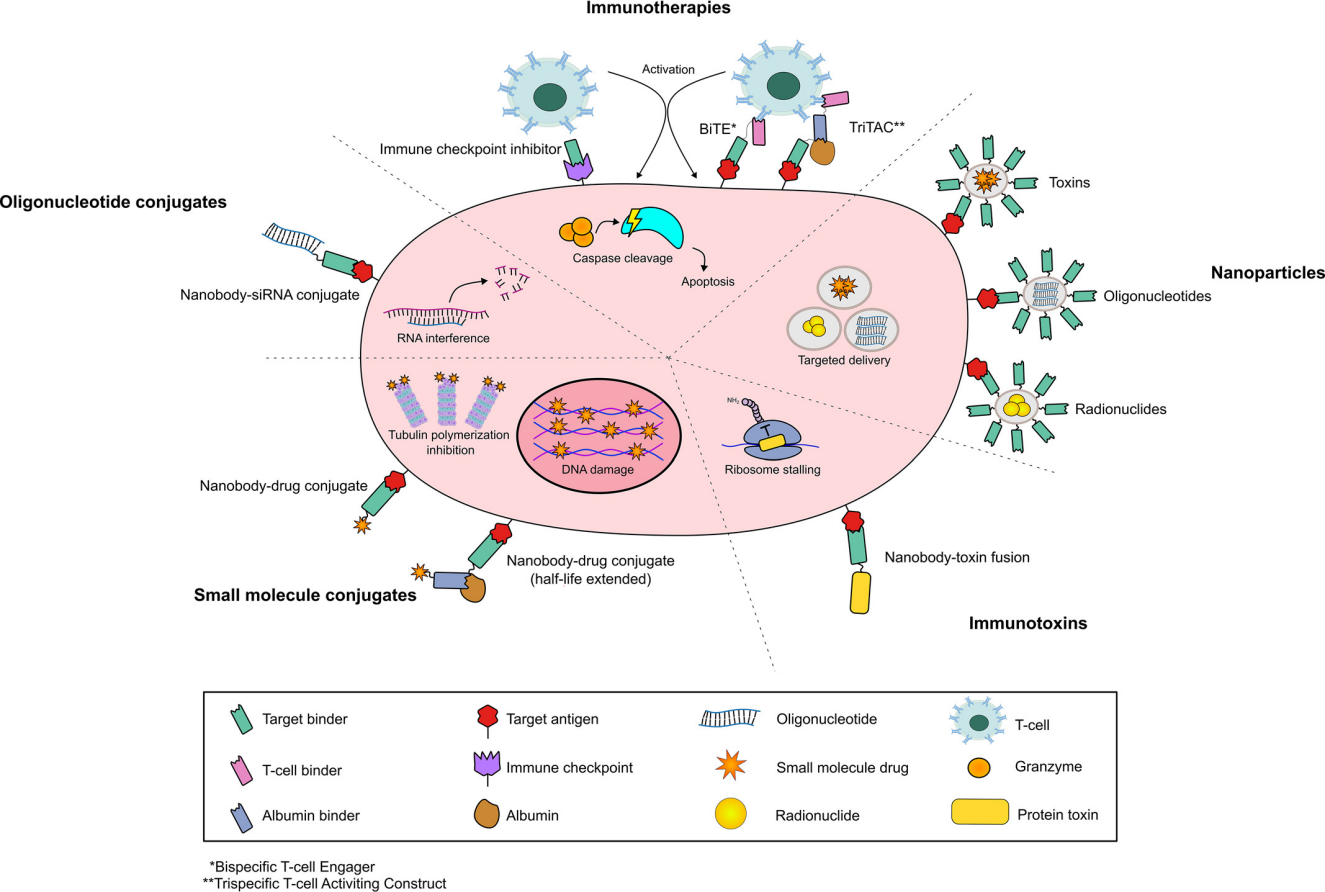
The biomedical applications of nanobodies. Schematic representation of nanobody-based modalities for therapy.

Beyond their use as molecular tools, nanobodies have shown substantial merit as building blocks for therapeutic agents (Fig. 5). As discussed in *Nanobody-Enzyme Fusion Proteins*, their ease of handling and biophysical robustness makes VHHs potent tools for chemical conjugation. Aside from their more commonplace conjugation to fluorophores, however, nanobodies can also be equipped with cytotoxic small molecules to produce much smaller variants of antibody-drug conjugates (ADCs). In this context, nanobody-drug conjugates (NDCs) are often touted for their enhanced tumor penetrance and biodistribution compared with ADCs owing to their extremely small relative size (131). However, NDCs are still largely underdeveloped in comparison with ADCs and indeed to other forms of nanobody conjugates, mainly due to the high renal clearance rates of proteins less than 40 kDa in size (132). Wu et al. (53) demonstrated the tradeoffs of NDC technology by generating a fully human VH with superior, camelid-like biophysical properties targeting the tumor-associated antigen 5T4. This construct, when conjugated to the topoisomerase inhibitor SN-38, outperformed its IgG-based ADC counterpart in subcutaneous tumor xenograft experiments. The improved efficacy was attributed to the NDC’s superior tumor accumulation and tissue penetration. This was elegantly supported by tumor spheroid experiments, wherein the sdAb could access the inner cell mass much more efficiently than the full-length antibody. However, the in vivo experiments necessitated more frequent dosing of the NDC compared with the ADC due to its suboptimal clearance rate, highlighting the main clinical roadblock in using nanobodies for tumor targeting. Many therefore deem it necessary that nanobody-based drugs are modified for stability in circulation. One of the most popular methods to achieve this is by genetically fusing the therapeutic nanobody to an albumin binder, allowing for the construct to “piggyback” onto serum albumin and thus remain in circulation for much longer. Many NDCs have used this method to varying degrees of success (133–135). Nessler et al. (134) directly compared the in vivo efficacy of a biparatopic NDC fusion with or without an albumin-binding domain (ABD), demonstrating rapid clearance, lower tumor accumulation, and overall suboptimal efficacy of the unfused NDC. Xenaki et al. (133) followed this in 2021, reporting a human epidermal growth factor receptor (HER2)-targeting NDC fused to a peptide-based ABD. Once again, dramatic increases (∼15-fold) in serum half-life combined with excellent cytotoxicity in vitro and in vivo were demonstrated with an albumin-stabilized NDC. The authors also reported deep-tissue penetration of the albumin-complexed NDC, emphasizing that the relatively large size of the complex does not incur a significant cost to tissue distribution. A similar, fully camelid, albumin-linked NDC-targeting trophoblast cell surface antigen 2 (TROP-2) showed extremely promising results within pancreatic cancer mouse models (135).

A more nascent modality is the use of nanobody-oligonucleotide conjugates. As opposed to antibodies, VHHs present a small, robust, and easy-to-handle scaffold to chemically attach bulky oligonucleotide payloads. The most success has been seen with nanobodies linked to siRNA, which allows for the specific knockdown of target genes through RNA interference (RNAi) (136). Zavoiura et al. (120) provided the clearest proof of principle for this strategy in 2021, conjugating half-life-extended EGFR-targeting nanobodies with siRNA payloads and tracking their efficient uptake into the cytosol. Ultimately, the authors achieved target mRNA knockdown of >60% specifically in antigen-positive cell populations. A less direct delivery mode was developed by Cunha-Santos et al. (137), where a nanobody targeting CXCR4 was fused to a FITC-targeting scFv. A FITC-conjugated siRNA targeting the Tat protein (an HIV transcriptional regulator) could then be loaded onto the fusion protein through the scFv. The construct was directed to T cells through the αCXCR4 nanobody, which culminated in an ∼80% reduction in Tat expression and viral replication.

### Nanobody-Based Immunotoxins

Although small molecule-based ADCs dominate the clinical landscape, another viable strategy lies in genetically fusing antibodies to cytotoxic protein domains. These agents, known as immunotoxins (ITXs), are generally lauded for circumventing a key concern associated with ADCs. Since ITXs are genetically fused, inter- and intrabatch heterogeneity is not an issue, in contrast to any chemically conjugated modality. In this application, smaller binding units such as scFvs and nanobodies are vastly preferred to full-length antibodies, which are far less likely to tolerate the addition of bulky protein domains. Nanobodies, on the other hand, can be relatively easily incorporated into fusion constructs whilst maintaining conformational stability and expressibility. The most used fusion partners for ITXs are the bacterial proteins pseudomonas exotoxin A (PE) and diphteria toxin (DT), both stalling the target cells’ translational machinery. Nanobody-based immunotoxins, although still not clinically advanced, have shown recent promise. Examples include ITXs targeting TROP2 (138), EGFR (139), and CD7 (140), all having delivered encouraging results in vivo.

### Multispecific Nanobodies in Immunotherapy

Having set a precedent as ideal constructs for protein-protein fusions, nanobodies have become tremendously useful in the construction of multispecific binders. While generating IgG-based bispecifics necessitates complicated and low-yield methods such as knob-into-hole assembly, nanobodies of different specificities can easily be fused on a genetic level. As such, numerous nanobody-based bispecifics exist in various stages of the clinical pipeline, frequently as bispecific T cell engagers (BiTEs). This modality, originally developed using scFvs, usually combines one binder against a tumor-associated antigen with another towards a T cell activating receptor (conventionally CD3) and was launched to the forefront of cancer immunotherapy by the clinical success of the FDA-approved Blinatumomab. Alternating from these conventional scFv fusions, nanobodies are well-embedded into the modern clinical landscape of BiTEs. For example, Yang et al reported a BiTE linking CD3 and CD105-targeting nanobodies which showed excellent T cell-mediated efficacy in hepatocellular carcinoma mouse models, whilst maintaining a favorable toxicity profile (141). However, the greatest novelty of nanobody-based BiTEs once again arises from their role as robust building blocks. The most prominent example of this is the trispecific T cell activating construct (TriTAC), developed by Harpoon Therapeutics (142). This modality comprises a TAA-binding nanobody fused to an αCD3 scFv in addition to a nanobody targeting human serum albumin. Primarily directed against solid tumors, TriTACs have proven an elegant strategy to maintain superior tissue penetration whilst overcoming serum stability roadblocks normally imparted by the small size of BiTEs. A pioneering study by Austin et al. (142) showed superb T cell mediated killing by TriTACs, with EC_50_ values consistently in mid-to-low picomolar ranges. This carried over in vivo, with doses as low as 2 µg/kg showing superb regression in subcutaneous tumor mouse models. Their effectiveness, coupled with their favorable pharmacokinetic and safety profiles in vivo (143), has culminated in a slew of TriTAC-based BiTEs currently in early clinical trials for various indications (143–145).

Beyond T cell engagement, nanobodies have also found use in other immunotherapeutic strategies such as immune checkpoint inhibition. Zhai et al. (146) generated an innovative nanobody-based bispecific, PM1003, targeting both the T cell costimulatory receptor 4-1BB and the immune checkpoint PD-L1. This dual-acting agent showed remarkable T cell mediated killing and immune memory induction, which was augmented by the simultaneous engagement of PD-L1. The authors also reported that the 4-1BB VHH accessed a membrane-proximal binding site that is distinct from other 4-1BB antibodies. They speculate that this binding site contributes to the clearly improved tolerability of PM1003, without compromising potency relative to other full-length antibodies. Whether access to this epitope was conferred by the unique binding characteristics of VHHs, or rather a serendipitous phenomenon, is unclear. Regardless, PM1003 represents yet another well-advanced nanobody-based immunotherapeutic, currently in phase I clinical trials. Another strategy was recently developed by Liu et al, where nanobodies targeting Cytotoxic T lymphocyte antigen 4 (CTLA-4) were fused to an Fc-targeting VHH (147). The latter nanobody binds to circulating host antibodies, providing both half-life extension as well as effector functions imparted by Fcγ-Receptor signaling. By accumulating antibodies in the tumor microenvironment and activating proximal macrophages via FcγR signaling, this construct could deplete intratumoral regulatory T cells. This resulted in the in vivo elimination of colorectal tumors by revitalizing immune activity at the tumor site. The authors further demonstrated nanobodies’ modular nature by using a binder targeting PD-L1 in lieu of CTLA-4 and conjugating the fusion protein with a small molecule drug, allowing the direct elimination of PD-L1^+^ tumor cells. Owing to nanobodies’ superb modularity, further array of singly- and multi-specific nanobody-based immune checkpoint inhibitors exist in various stages of clinical progress, alongside other agents such as chimeric antigen receptor T (CAR-T) cells and natural killer (NK) cell engagers. A wide breadth of these is covered thoroughly in a recent review by Li et al. (148).

### Nanobodies for Targeted Delivery

A growing focus has been put on drug delivery via packaged nanoparticles, not least due to the recent successes of mRNA-based SARS-CoV-2 vaccines. Although prophylactic vaccines do not require targeted delivery per se, the combination of such packaging technologies with targeting platforms remains a captivating prospect in therapeutic development. Once again, initial strategies of nanoparticle targeting used full-length antibodies coupled, often stochastically, to the particle surface. Such methods resulted in the successful delivery of small interfering RNA (siRNA) (149, 150), mRNA (151–154), and small molecules (155) to target cells. These agents can, however, prove challenging to manufacture. Obstacles include inefficient assembly/conjugation, batch heterogeneity, nanoparticle instability, and suboptimal intracellular escape (149, 156). Antibody fragments, such as scFvs, have been used to partially alleviate some of these drawbacks, with many recent examples of effective, efficiently conjugated scFv-functionalized nanoparticles (149, 157). Nanobodies, sidestepping the inherent stability issues of scFvs, represent a potential tool to boost targeted nanoparticle technology further. Noh et al. (158) recently established a delivery system using a high-affinity nanobody targeting CD155, a lung adenocarcinoma target associated with lymphocyte inhibition, tumor migration, and poor prognosis. In this study, doxorubicin-loaded DPPC-based liposomes were conjugated to the thiolated nanobody through interspersed DSPE-maleimide monomers. This resulted in the efficient decoration of the nanoparticles while maintaining nearly identical size, polydispersity, and surface zeta potential to the unmodified liposomes, attesting to nanobodies’ suitability for unperturbed nanoparticle targeting. The nanobody-liposome complexes maintained pH-mediated drug release, showed threefold improved targeting, and demonstrated drastically improved in vitro and in vivo performance compared with undecorated liposomes and bare nanobodies in lung adenocarcinoma models. Rahman et al. presented an innovative strategy, wherein HER2-specific nanobodies fused to a C-terminal single transmembrane domain (STMD) were generated (159). These nanobodies could thus spontaneously embed themselves into the lipid bilayer of a liposome via the STMD, achieving remarkable decoration levels of up to ∼2,500 nanobodies per nanoparticle. This vehicle was subsequently used to deliver 5-fluorouracil to HER2+ cells in vitro and in vivo. Nanobodies also serve as exciting tools for modular docking-based delivery systems. Chen et al. (156) elegantly demonstrated this when they generated a high-affinity nanobody targeting the Fc region of murine IgGs. This nanobody, TP1107, was carefully examined for its paratope to ensure an outward-facing orientation of the nanobody-Fc complex when conjugated. TP1107 was site specifically conjugated to DSPE-PEG_2000_ through strain-promoted click chemistry and incorporated into mRNA-loaded lipid nanoparticles (LNPs) at a rate of ∼200 nanobodies per particle, maintaining favorable biophysical characteristics. As a result, the authors were able to couple a variety of full-length mAbs to the TP1107-decorated LNPs with quantitative efficiency and confer extremely efficient targeting toward the T cell antigens CD7, CD5, CD4, and CD3, the latter of which was successfully used in vivo with minimal safety concerns. Although the authors used enhanced green fluorescent protein (eGFP) mRNA as a model cargo, this study represents an exciting leap toward specific therapeutic mRNA delivery in a fully modular manner.

## CONCLUSIONS

The observation of hcAbs in camelids roughly 30 yr ago was as serendipitous as it was revolutionizing. Through the simple absence of a pairing VL domain, nanobodies have emerged as an indispensably unique item in the catalog of antibody formats. Although traditional antibodies will likely remain at the forefront for established modalities such as antibody-drug conjugates, nanobodies have been able to perform outstandingly in a niche of previously underexplored applications. This is reflected in the continually growing body of work, wherein nanobodies have been used to explore cellular physiology, an application seemingly limited only by the scope of available fusion partners. The widespread adoption of nanobodies for next-generation imaging applications and their latent biomedical use as drug conjugates, immunotherapeutics, and drug delivery agents, all but ensures that nanobodies will continue their supremely impressive trajectory over the next years.
